# Pathogen aetiology and risk factors for death among neonates with bloodstream infections at lower-tier South African hospitals: a cross-sectional study

**DOI:** 10.1016/j.lanmic.2024.100989

**Published:** 2025-05

**Authors:** Susan Meiring, Vanessa Quan, Rudzani Mashau, Olga Perovic, Rindidzani Magobo, Marshagne Smith, Ruth Mpembe, Anne von Gottberg, Linda de Gouveia, Sibongile Walaza, Cheryl Cohen, Constance Kapongo, Cheryl Mackay, Mphekwa Thomas Mailula, Omphile Mekgoe, Lerato Motjale, Rose Phayane, Angela Dramowski, Nelesh P Govender, Baby GERMS-SA, Danie Erwee, Dina Pombo, Erika van Schalkwyk, Greta Hoyland, Harishia Naidoo, Juliet Paxton, Lesley Ingle, Linda Erasmus, Meluleki Mthimkhulu, Ntombi Dube, Phunyezwa Mzayiya, Ramatlhwa Kekana, Ruth Lekalakala, Sandra Maphumulo, Sebabatso Khantsi, Shareef Abrahams, Sindile Ntuli, Siyanda Dlamini, Thembekile Zwane, Thulisile Maphosa, Vanessa Pearce

**Affiliations:** a National Institute for Communicable Diseases, a Division of the National Health Laboratory Service, Johannesburg, South Africa; b Faculty of Health Sciences, University of the Witwatersrand, Johannesburg, South Africa; c Faculty of Health Sciences, University of Cape Town, Cape Town, South Africa; d Department of Paediatrics and Child Health, Queen Nandi Regional Hospital, Ngwelazane, South Africa; e Department of Paediatrics and Child Health, Dora Nginza Hospital, Nelson Mandela Bay, South Africa; f Department of Paediatrics and Child Health, Mankweng Regional Hospital, Mankweng, South Africa; g Department of Paediatrics and Child Health, Klerksdorp Regional Hospital, Klerksdorp, South Africa; h Department of Paediatrics and Child Health, Rob Ferreira Regional Hospital, Nelspruit, South Africa; i Department of Paediatrics and Child Health, Tembisa Provincial Hospital, Johannesburg, South Africa; j Department of Paediatrics and Child Health, Faculty of Medicine and Health Sciences, Stellenbosch University, Cape Town, South Africa; k MRC Centre for Medical Mycology, University of Exeter, Exeter, UK

## Abstract

**Background:**

Infections are among the top causes of neonatal mortality, particularly in low-income and middle-income countries. We aimed to describe the clinical characteristics of neonates diagnosed with culture-confirmed bloodstream infections at six lower-tier hospitals in South Africa.

**Methods:**

We did a cross-sectional study of culture-confirmed bloodstream infections among neonates (aged 0–27 days) at six lower-tier hospitals in South Africa. Clinical, demographic, and pathogen data from sick, hospitalised neonates were analysed and bloodstream infections were categorised as early-onset sepsis (EOS; 0–2 days of life) or late-onset sepsis (LOS; 3–27 days of life). Incidence of bloodstream infection and crude in-hospital mortality in neonates with bloodstream infection were calculated and factors associated with death were analysed using multivariable logistic regression models.

**Findings:**

From Oct 1, 2019 to Sept 30, 2020, we identified 907 neonatal bloodstream infection episodes. Incidence was 6·4 cases per 1000 patient-days. Most neonates were preterm (median gestation 33 weeks [IQR 29–37]), with 30·5% (n=277) of bloodstream infections classified as EOS and 69·5% (n=630) as LOS. Gram-negative pathogens dominated (63·2% [n=573]), including *Klebsiella pneumoniae* (25·7% [n=233]) and *Acinetobacter baumannii* (19·2% [n=174]). Crude in-hospital mortality in neonates with bloodstream infection was 25·5% (n=231), accounting for 21·4% (231 of 1078 cases) of all in-hospital neonatal deaths. Increased all-cause mortality was associated with Gram-negative bloodstream infection (*vs* Gram-positive pathogens, adjusted odds ratio 3·70 [95% CI 1·46–9·39]; p=0·0059), inborn LOS (*vs* EOS, 2·42 [1·11–5·29];  p=0·027), preterm birth (5·00 [2·16–11·59]; p=0·0002), and neonatal intensive care unit admission (3·26 [1·51–7·03]; p=0·0026).

**Interpretation:**

Hospitalised, preterm neonates who developed Gram-negative bloodstream infections had high in-hospital mortality. Many small vulnerable newborns require prolonged stays in lower-tier hospitals and acquire life-threatening bloodstream infection; appropriate resources are needed at this level of care to prevent infections and save lives.

**Funding:**

Bill & Melinda Gates Foundation.

## Introduction

Infectious diseases are among the top three causes of neonatal mortality, particularly in low-income and middle-income countries where 99% of global neonatal deaths occur.^1^^,^^2^ Sustainable Development Goal 3.2 is to reduce neonatal mortality to fewer than 12 deaths per 1000 livebirths by 2030, an ambitious target for the African continent, which in 2021 recorded a rate of 26 deaths per 1000 livebirths.^2^ Globally, an estimated 3 million episodes of neonatal sepsis (22 episodes per 1000 livebirths) occur annually, with an estimated incidence 40 times higher in middle-income countries than high-income countries.^3^

**Research in context**

**Evidence before this study**

We searched Google Scholar and PubMed for research publications from Jan 1, 2000, to Nov 30, 2024, using the search terms “neonate” OR “newborn” AND “sepsis” OR “bloodstream infection” OR “invasive infection” OR “bacteraemia”. Infections are among the top three causes of neonatal mortality, with most infections and deaths among neonates reported in middle-income and low-income countries. The few studies conducted in Africa report on neonatal sepsis at academic or tertiary institutions and most confine their findings to either health-care-associated infections or early-onset sepsis. A population-based study on the incidence risk of neonatal infections in the South African public sector described an increase in neonatal bloodstream infections between 2014 and 2019, with the majority of these occurring in lower-tier hospitals. Understanding the burden and aetiology of infections at lower-tier hospitals is essential to guide resource allocation and public health interventions to prevent infections.

**Added value of this study**

This observational study from an upper-middle-income country highlights the high incidence and attributable mortality of neonatal bloodstream infections in lower-tier hospitals. Gram-negative pathogens dominate, with *Klebsiella pneumoniae* causing most early-onset and late-onset infections. In addition, two-thirds of the pathogens causing bloodstream infections were resistant to three or more drug classes, with over 80% of *Acinetobacter baumannii* isolates being carbapenem resistant. Most neonatal bloodstream infections occurred in preterm babies who required prolonged hospital stays, highlighting the need for adequate resources to care for the many small vulnerable newborns in lower-tier hospitals.

**Implications of all the available evidence**

There is a high burden of neonatal sepsis occurring among preterm neonates in lower-tier hospitals. Policy makers in middle-income and lower-income countries should ensure that lower-tier hospitals are adequately resourced to care for and prevent severe infections in small vulnerable newborns. The dominance of Gram-negative pathogens in both early-onset and late-onset sepsis, coupled with the high rate of multidrug resistance among the top three causative pathogens, highlights the need for re-evaluation of WHO-recommended first-line and second-line empirical antibiotic regimens and development of site-specific antibiograms.

Bloodstream infections, of which only a portion are culture confirmed, are an important cause of sepsis in hospitalised neonates. A comprehensive understanding of the characteristics of neonates with bloodstream infections and the pathogen distribution at all levels of health care would facilitate resource allocation and better planning of health interventions to prevent infections.^3^ In South Africa, public-sector hospitals are classified as district, regional, provincial, central, or specialised, with increasing levels of care and means for referral. A South African population-based surveillance study reported a national incidence risk of culture-confirmed neonatal bloodstream infection and meningitis of six cases per 1000 livebirths (1·1 per 1000 livebirths for early-onset sepsis [EOS] and 4·9 per 1000 livebirths for late-onset sepsis [LOS]).^4^ A previous study showed that more than 70% of laboratory-confirmed neonatal infections (bloodstream infection and meningitis) were diagnosed in lower-tier (ie, district, regional, and provincial) hospitals. Despite the high burden of neonatal infections in these lower-tier hospitals, most studies from Africa have exclusively reported data from central referral hospitals, which might have a different bloodstream infection incidence and cause.

*Klebsiella pneumoniae* and *Acinetobacter baumannii* are the dominant causes of neonatal infections in low-income and middle-income countries.^5^, ^6^, ^7^, ^8^ Of concern is the declining susceptibility of these Gram-negative bacteria to many antibiotic classes, including empirical antibiotic regimens recommended by WHO.^9^ Studies from large referral hospitals show that most Gram-negative infections are late-onset and occur among preterm infants with prolonged hospital stays.^5^^,^^8^ Mashau and colleagues showed that Gram-positive neonatal infections were relatively more common in lower-tier versus higher-tier hospitals and both Gram-negative and Gram-positive bacteria were generally less resistant to antimicrobials in lower-tier hospitals.^4^

Neonates in lower-tier hospitals might be inherently different from those managed in the highest tiers of hospital care in terms of their gestational age, HIV exposure, and empirical antibiotic therapy.^4^ In this enhanced surveillance study, we aimed to describe the clinical characteristics of neonates diagnosed with culture-confirmed bloodstream infection at six lower-tier hospitals in South Africa, including the distribution and antimicrobial susceptibility profile of bloodstream infection pathogens and the factors associated with neonatal death.^10^

## Methods

### Study design and participants

This cross-sectional study was conducted in South Africa, an upper-middle-income country, in which 91% of births are attended by skilled health personnel in a health-care facility.^11^ In 2019, there were 1 178 116 registered livebirths, with a neonatal mortality rate of 11 in 1000 livebirths.^12^ The rate of preterm births (<37 weeks of gestation) was estimated to be 12 of 1000 livebirths.^13^ In 2019, the HIV prevalence among antenatal clinic attendees was 30%, with less than 1% of children acquiring HIV infection by vertical transmission.^14^

This study was nested within a national population-based surveillance study on culture-confirmed neonatal infections at all public-sector hospitals in South Africa (Baby GERMS-SA), and the protocol for both studies has been published.^10^ We conducted a cross-sectional study among neonates with culture-confirmed bloodstream infection between Oct 1, 2019, and Sept 30, 2020, admitted to six lower-tier hospitals (three provincial and three regional or district hospitals; appendix p 4).^10^ Clinicians ordered blood cultures on clinically unwell neonates at their discretion and the diagnostic pathology laboratories submitted isolates from any positive blood cultures on Dorset agar medium (Diagnostic Media Products, Johannesburg, South Africa) to the National Institute for Communicable Diseases (NICD) for further characterisation. The study was reported using the STROBE-NI guideline.^15^

The study was approved by the Human Research Ethics Committee of the University of the Witwatersrand (M190320). Informed consent for the retrospective collection of clinical data was not required for this observational study (as confirmed by the ethics committee); therefore, medical record reviews were performed after neonates had been discharged from hospital and no personal identifiers were collected on the database.

### Procedures

The total number of neonatal admissions, bed-days, and in-hospital deaths were collected from each hospital’s administration department. Aggregated total numbers of blood cultures performed on all neonates at the six hospitals and a line list of individual neonates with positive blood cultures were obtained from the NICD Surveillance Data Warehouse. Medical charts from neonates with a bloodstream infection were imaged and clinical data were abstracted by medical officers, trained in data abstraction, onto a REDCap database.^16^ Clinical data included sex, date of birth, gestational age and birthweight, current age and weight at time of blood culture, antibiotic receipt, neonatal intensive care unit admission, admission and outcome date, in-hospital outcome, and HIV status of the mother and infant.

An episode of neonatal bloodstream infection was defined when a pathogenic organism was identified from the blood culture of a neonate (aged 0–27 days) admitted to one of the six hospitals. Pathogens and commensals were defined according to the US Centers for Disease Control and Prevention classification. Coagulase-negative *Staphylococcus* was defined as a pathogen only when isolated from two or more blood cultures taken at separate times within a 2-day period. Subsequent positive blood cultures from the same neonate taken within 14 days of an initial positive culture were counted within the same episode and further defined as a monomicrobial episode if a single species was cultured or as a polymicrobial episode if more than one species was cultured. Organisms from polymicrobial episodes were enumerated separately and considered independent when describing pathogen characteristics.

Neonates with culture-positive bloodstream infection were categorised into those with EOS (0–2 days of life) and LOS (3–27 days of life) episodes. Neonates with LOS were further categorised into those who had remained in hospital since birth (inborn LOS) and those readmitted from the community after post-birth discharge (readmitted LOS). Neonates were considered preterm if they were born before 37 weeks of gestation (based on either the date of mother's last menstrual period or symphysis-to-fundal height measurement during antenatal care). The WHO subdivision of preterm birth was used to further categorise gestational age as term (born after 37 completed weeks of gestation), moderate preterm (33–36 weeks of gestation), very preterm (28–32 weeks of gestation), or extremely preterm (<28 weeks of gestation).

WHO recommends a combination of ampicillin–benzylpenicillin plus gentamicin as first-line treatment of neonatal sepsis, and third-generation cephalosporins or cloxacillin plus amikacin as second-line treatment options.^17^ In South Africa, most neonatal units use piperacillin–tazobactam plus amikacin as the empirical second-line antibiotic regimen for suspected LOS and health-care-associated infection, with meropenem (third-line treatment) reserved for suspected meningitis or life-threatening infections.^18^ We report on the antimicrobial susceptibility of Gram-negative and Gram-positive pathogens to these empirical antibiotic regimens for LOS. For combination therapies, pathogens were considered resistant if they were resistant to both antibiotics. Neonates still in hospital on day 28 of life were considered to have survived the bloodstream infection episode, otherwise neonatal outcome was determined at the time of hospital discharge or death. Any neonatal death occurring within 3 calendar days of a positive blood culture was attributed to the sepsis episode as the immediate cause of death.^19^

Diagnostic microbiology laboratories all used automated blood culture systems and methods, such as Vitek-2 (bioMerieux, Marcy-l'Etoile, France), Microscan Walkaway (Beckman Coulter, Brea, CA, USA), or Vitek MS (bioMerieux, Marcy-l'Etoile, France) to identify bacterial and fungal pathogens and perform antimicrobial susceptibility testing. At the NICD, isolate identification was confirmed using standardised methods accredited to ISO15189:2021 by the South African National Accreditation System. Minimum inhibitory concentrations (MICs) for indicating antimicrobial susceptibility were generated on a MicroScan instrument (Beckman Coulter, West Sacramento, CA, USA) using Microscan GNX2F, Neg MIC Type 44, or Positive MIC Type 33 panels (Thermo Fisher Scientific, Waltham, MA, USA), or manually using Sensititre STP6F (*Streptococcus* spp) or YeastOne Y010 (*Candida* spp) panels (Thermo Fisher Scientific). MICs were interpreted according to the Clinical and Laboratory Standards Institute recommendations. Isolates were classified as multidrug-resistant if they were non-susceptible to one or more agents in two or more antimicrobial classes as defined by an international expert committee.^20^ Details of isolate culture, identification, and antibiotic susceptibility testing are available in the appendix (p 3).

### Outcomes

The primary outcome of the study was to examine the distribution of pathogens causing bloodstream infection episodes in neonates. The secondary outcomes were to determine associations between various risk factors and death after neonatal bloodstream infection episodes, as well as associations related to the timing of the sepsis episode.

### Statistical analysis

Descriptive statistics were reported as frequencies for categorical variables and medians with IQRs for continuous variables. Analytical tests for association between categorical and non-parametric numerical variables were done using the Wilcoxon rank sum test. Rates of blood culture specimen collection per admitted neonate were reported, as well as incidence rates of bloodstream infection expressed separately per 1000 patient-days and per 1000 livebirths. In-hospital mortality risk was calculated among all admitted neonates, including the crude (all deaths) and attributable (deaths within 3 days of sepsis episode) case-fatality ratios among neonates with bloodstream infection. Univariable and multivariable logistic regression models were performed to determine odds ratios (ORs) and 95% CIs for variables associated with in-hospital death by day 27 of life. Variables with a p value of <0·2 on univariate analysis were included in all multivariable logistic regression models, and those with a p value of >0·05 were then dropped using step-wise manual backward elimination. p values <0·05 were considered significant. Three variables were included a priori in the models: gestational age, admission to an intensive care unit, and empirical antibiotic therapy concordant with the isolated pathogen’s susceptibility. Observations with missing data were excluded from the final multivariable analysis. Additional univariate analyses (exploring pathogen type and antimicrobial susceptibility) were conducted to determine associations with timing of the sepsis episode. All statistical analyses were performed using STATA statistical software version 17.

### Role of the funding source

The funder had no role in the study design, data collection, data analysis, data interpretation, or writing of the report.

## Results

From Oct 1, 2019, to Sept 30, 2020, 38 387 blood cultures were submitted for investigation of suspected sepsis from 23 381 neonates at six hospitals (rate of 1·6 blood cultures per admitted neonate). 2613 (6·8%) specimens were culture positive, of which 1469 (56·2%) were commensals (including 1234 single coagulase-negative *Staphylococcus* spp; figure). Among 1028 pathogenic organisms, 907 (88·2%) discrete episodes of neonatal bloodstream infection were identified: 801 monomicrobial and 106 polymicrobial (appendix pp 6–7).

At lower-tier hospitals, the incidence rate of neonatal bloodstream infection was 4·9 episodes per 1000 livebirths in the districts served (1·5 episodes of EOS and 3·4 episodes of LOS per 1000 livebirths). Of all 23 381 hospitalised neonates, there were 907 (3·9%) diagnosed bloodstream infections. The median onset of bloodstream infection was day 6 of life (IQR 1–11), with 277 (30·5%) of the 907 bloodstream infections classified as EOS and 630 (69·5%) LOS. Among 856 bloodstream infection episodes in neonates with hospital admission dates available, LOS was further characterised as LOS among inborn neonates (473 [55·3%]) and LOS among readmitted neonates (106 [12·4%]). The incidence rate of all neonatal bloodstream infections, EOS, inborn LOS, and readmitted LOS was 6·4 cases per 1000 patient-days, 2·1 cases per 1000 patient-days, 3·5 cases per 1000 patient-days, and 0·8 cases per 1000 patient-days, respectively. Among all admitted neonates, there were 1078 neonatal deaths (56 among those with EOS, 175 with LOS, and 847 probably unrelated to a bloodstream infection). Crude in-hospital mortality was 4·6% (1078 of 23 381 neonates) among all admitted neonates, 3·8% (847 of 22 474 neonates) among those without a diagnosed bloodstream infection, and 25·5% (231 of 907) in culture-confirmed bloodstream infection episodes. Of all 1078 neonatal deaths, 231 (21·4%) occurred in those with a laboratory-confirmed bloodstream infection.

Clinical data were available for 895 (98·7%) of 907 neonatal bloodstream infection episodes; bloodstream infection episodes were distributed evenly by hospital type (446 [49·2%] provincial *vs* 461 [50·8%] regional or district; table 1). 486 (70·0%) of 694 neonates with gestational age data were preterm. The median gestational age of neonates was 33 weeks (IQR 29–37) and their median birthweight was 1670 g (IQR 1150–2670). 487 (56·5%) of 862 neonates with available data were male. 235 (34·1%) of 690 neonates with available data were HIV exposed (appendix p 9); four (2·0% of 204) HIV-exposed neonates with available PCR test results were later confirmed to have HIV infection.

**Table 1:** Characterisation of episodes of laboratory-confirmed neonatal bloodstream infections (n=907)

	Number of cases or median
**Hospital site characteristics**	
Hospital type∗	
Regional or district	461/907 (50·8%)
Provincial	446/907 (49·2%)
Hospital name	
Dora Nginza	143/907 (15·8%)
Klerksdorp	116/907 (12·8%)
Mankweng	237/907 (26·1%)
Queen Nandi	128/907 (14·1%)
Rob Ferreira	93/907 (10·3%)
Tembisa	190/907 (20·9%)
**Neonatal case characteristics**	
Sex	
Female	375/862 (43·5%)
Male	487/862 (56·5%)
Median gestational age, weeks	33 (29–37)
Gestation category	
Term	208/694 (30·0%)
Preterm	486/694 (70·0%)
Moderate (33–36 weeks)	167/694 (24·1%)
Very (28–32 weeks)	254/694 (36·6%)
Extreme (<28 weeks)	65/694 (9·4%)
Median birthweight, g	1670 (1150–2670)
HIV exposure status	
Mother HIV-infected	235/690 (34·1%)
Mother HIV-uninfected	455/690 (65·9%)
HIV infection status	
Neonates with HIV infection†	4/622 (0·6%)
Neonates without HIV infection‡	618/622 (99·4%)
HIV mother-to-child transmission rate	4/204 (2·0%)
**Neonatal bloodstream infection episode characteristics**	
Median age at date of culture, days	6 (1–11)
Median admission days at date of culture, days	4 (0–9)
Presentation (timing of sepsis onset)	
EOS	277/907 (30·5%)
LOS	630/907 (69·5%)
Inborn LOS	473/907 (52·1%)
Readmitted LOS	106/907 (11·7%)
Admission date unknown	51/907 (5·6%)
Antimicrobial therapy received	
Ampicillin–penicillin plus gentamicin	142/535 (26·5%)
Piperacillin–tazobactam plus amikacin	87/535 (16·3%)
Carbapenem	180/535 (33·6%)
Third-generation cephalosporin	41/535 (7·7%)
Other	85/535 (15·9%)
Outcome by day 27 of life	
Survived	590/792 (74·5%)
Died	202/792 (25·5%)
Median duration of hospitalisation, days	16 (7–31)
Time to discharge	20 (11–37)
Time to death	7 (4–14)
Type of infection	
Gram-negative pathogen	573/907 (63·2%)
Gram-positive pathogen	285/907 (31·4%)
Fungal pathogen	49/907 (5·4%)

Data are n/N (%) or median (IQR). Denominators different from 907 represent cases with data available. No data on race or ethnicity were collected in this study.

∗ There were no significant differences in proportion of term versus preterm neonates (p=0·64), nor proportion of episodes of early-onset *vs* late-onset sepsis (p=0·98), by hospital type (provincial *vs* regional or district).

† Confirmed by PCR.

‡ Confirmed by PCR or ELISA. EOS=early-onset sepsis (0–2 days of life). LOS=late-onset sepsis (3–27 days of life).

Of the 544 isolates sent to the NICD reference laboratory for confirmation and further characterisation, only eight (1·5%) had been misidentified at genus or species level by the diagnostic laboratory (figure). A comparison of antimicrobial susceptibility test results generated by the diagnostic and reference laboratories is available in the appendix (p 8).

**Figure:**
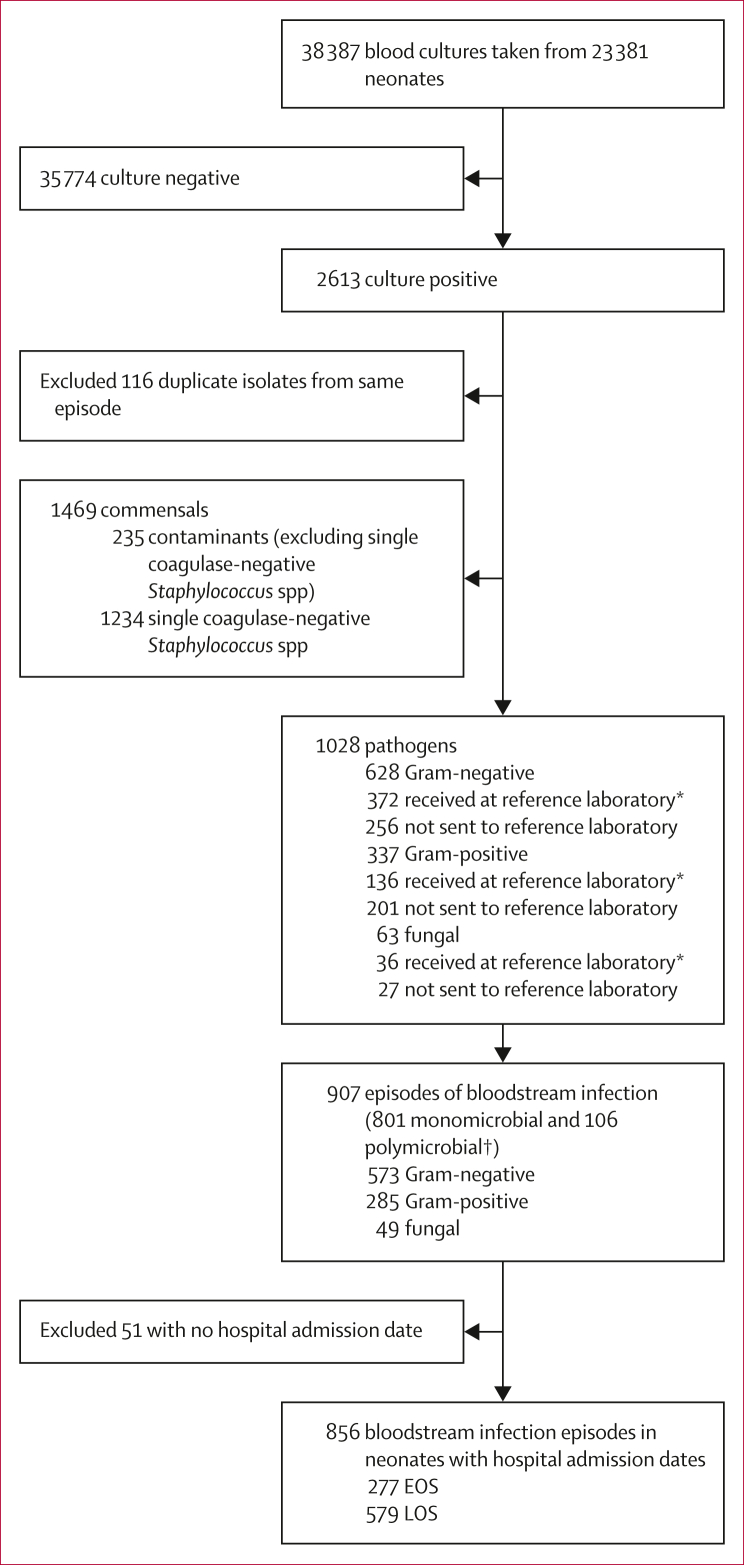
Study profile ∗Eight of 544 had discordant identification to the clinical laboratory. †106 polymicrobial episodes comprised one bloodstream infection episode of five pathogens, one episode of four pathogens, nine episodes of three pathogens, and 96 episodes of two pathogens. EOS=early-onset sepsis (0–2 days of life). LOS=late-onset sepsis (3–27 days of life).

Of all 907 bloodstream infection episodes, 573 (63·2%) were caused by Gram-negative bacteria, 285 (31·4%) by Gram-positive bacteria, and 49 (5·4%) by fungi. 604 (66·6%) of all 907 episodes of bloodstream infection were caused by five bacteria—namely, *K pneumoniae* (233 [25·7%]), *A baumannii* (174 [19·2%]), *Staphylococcus aureus* (82 [9·0%]), *Enterococcus faecium* (59 [6·5%]), and *Streptococcus agalactiae* (56 [6·2%]; table 2). *K pneumoniae* was the most common pathogen among 277 episodes of EOS (41[14·8%]), 473 episodes of LOS among inborn neonates (156 [33·0%]), and 106 episodes of LOS among readmitted neonates (22 [20·8%]). Overall, *S aureus* was the most common Gram-positive pathogen (82 [28·8%] of 285 episodes of bloodstream infection with Gram-positive bacteria). *S agalactiae* was the second most common pathogen among 277 episodes of EOS (38 [13·7%]; appendix p 17). *Candida parapsilosis* was the most frequently isolated fungal pathogen (21 [42·9%] among 49 episodes of fungal bloodstream infection), particularly in EOS (12 [70·6%] of 17 episodes). Eight cases of *Candida auris* bloodstream infection were recorded.

**Table 2:** Aetiology of the top five neonatal bloodstream infections occurring at lower-tier hospitals by timing of presentation (n=907)

	All bloodstream infections	EOS	LOS	Inborn LOS (HAI)	Readmitted LOS
	(N=907)	(N=277)	(N=579)	(N=473)∗	(N=106)∗
Top five pathogens	604/907 (66·6%)	158/277 (57·0%)	422/579 (72·9%)	363/473 (76·7%)	70/106 (66·0%)
*Klebsiella pneumoniae*	233/907 (25·7%)	41/277 (14·8%)	178/579 (30·7%)	156/473 (33·0%)	22/106 (20·8%)
*Acinetobacter baumannii*	174/907 (19·2%)	33/277 (11·9%)	126/579 (21·8%)	113/473 (23·9%)	13/106 (12·3%)
*Staphylococcus aureus*	82/907 (9·0%)	27/277 (9·7%)	51/579 (8·8%)	34/473 (7·2%)	17/106 (16·0%)
*Enterococcus faecium*	59/907 (6·5%)	19/277 (6·9%)	37/579 (6·4%)	33/473 (7·0%)	..
*Streptococcus agalactiae*	56/907 (6·2%)	38/277 (13·7%)	..	..	11/106 (10·4%)
*Enterobacter cloacae*	..	..	30/579 (5·2%)	27/473 (5·7%)	..
*Enterococcus faecalis*	..	..	..	..	7/106 (6·6%)
Other organisms	303/907 (33·4%)	119/277 (43·0%)	157/579 (27·1%)	110/473 (23·3%)	36/106 (34·0%)
Top five Gram-negative pathogens	510/573 (89·0%)	106/140 (75·7%)	371/395 (93·9%)	320/337 (95·0%)	51/58 (87·9%)
*Klebsiella pneumoniae*	233/573 (40·7%)	41/140 (29·3%)	178/395 (45·1%)	156/337 (46·3%)	22/58 (37·9%)
*Acinetobacter baumannii*	174/573 (30·4%)	33/140 (23·6%)	126/395 (31·9%)	113/337 (33·5%)	13/58 (22·4%)
*Enterobacter cloacae*	43/573 (7·5%)	13/140 (9·3%)	30/395 (7·6%)	27/337 (8·0%)	3/58 (5·2%)
*Escherichia coli*	35/573 (6·1%)	13/140 (9·3%)	18/395 (4·6%)	8/337 (2·4%)	10/58 (17·2%)
*Serratia marcescens*	25/573 (4·4%)	6/140 (4·3%)	19/395 (4·6%)	16/337 (4·7%)	3/58 (5·2%)
Other Gram-negative organisms	63/573 (11·0%)	34/140 (24·3%)	24/395 (6·1%)	17/337 (5·0%)	7/58 (12·1%)
Top five Gram-positive pathogens	261/285 (91·6%)	107/120 (89·2%)	145/154 (94·2%)	102/108 (94·4%)	43/46 (93·5%)
*Staphylococcus aureus*	82/285 (28·8%)	27/120 (22·5%)	51/154 (33·1%)	34/108 (31·5%)	17/46 (37·0%)
*Enterococcus faecium*	59/285 (20·7%)	19/120 (15·8%)	37/154 (24·0%)	33/108 (30·6%)	4/46 (8·7%)
*Streptococcus agalactiae*	56/285 (19·6%)	38/120 (31·7%)	18/154 (11·7%)	7/108 (6·5%)	11/46 (23·9%)
*Enterococcus faecalis*	39/285 (13·7%)	16/120 (13·3%)	23/154 (14·9%)	16/108 (14·8%)	7/46 (15·2%)
Coagulase-negative *Staphylococcus*	25/285 (8·8%)	7/120 (5·8%)	16/154 (10·4%)	12/108 (11·1%)	4/46 (8·7%)
Other Gram-positive organisms	24/285 (8·4%)	13/120 (10·8%)	9/154 (5·8%)	6/108 (5·6%)	3/46 (6·5%)
Top five fungal pathogens	42/49 (85·7%)	14/17 (82·4%)	26/30 (86·7%)	25/28 (89·3%)	1/2 (50·0%)
*Candida parapsilosis*	21/49 (42·9%)	12/17 (70·6%)	8/30 (26·7%)	8/28 (28·6%)	..
*Candida albicans*	7/49 (14·3%)	2/17 (11·8%)	4/30 (13·3%)	4/28 (14·3%)	..
*Candida auris (haemulonii)*	8/49 (16·3%)	..	8/30 (26·7%)	7/28 (25·0%)	1/2 (50·0%)
*Candida famata*	4/49 (8·2%)	..	4/30 (13·3%)	4/28 (14·3%)	..
*Candida tropicalis*	2/49 (4·1%)	..	2/30 (6·7%)	2/28 (7·1%)	..
*Candida* species	..	1/17 (5·9%)	..	..	..
*Rhodotorula* species	..	1/17 (5·9%)	..	..	..
Yeasts	..	1/17 (5·9%)	..	..	..
Other fungal infections	7/49 (14·3%)	0/17 (0·0%)	4/30 (13·3%)	3/28 (10·7%)	1/2 (50·0%)

Data are n/N (%). EOS=early-onset sepsis (0–2 days of life). Inborn LOS HAI=late-onset sepsis hospital-associated infection among neonates who have never left the hospital since birth. LOS=late-onset sepsis (3–27 days of life). Readmitted LOS=late-onset sepsis among neonates admitted from home. ..=pathogen was not included in the top five organisms for that category.

∗ 51 neonates with LOS had unknown hospital admission dates and could not be characterised as inborn nor readmitted LOS.

Neonates presenting with EOS were significantly more likely to be infected with *S agalactiae* (p<0·0001), *Enterococcus faecalis* (p=0·0040), *Escherichia coli* (p=0·021), *S aureus* (p=0·015), or *E faecium* (p=0·037) than neonates with LOS.

Among Gram-negative organisms, 306 (59·2%) of 517 were resistant to both ampicillin–penicillin and gentamicin, 325 (66·2%) of 491 were resistant to third-generation cephalosporins, 151 (31·6%) of 478 were resistant to piperacillin–tazobactam plus amikacin, and 192 (38·9%) of 493 were resistant to carbapenems (table 3). Gram-negative organisms causing inborn LOS were three times more likely to be resistant to ampicillin–penicillin plus gentamicin (OR 3·1 [95% CI 2·1–4·6]; p<0·0001), twice more likely to be resistant to piperacillin–tazobactam and amikacin (1·9 [1·2–3·0]; p<0·011), and twice more likely to be resistant to carbapenems (2·0 [1·3–3·1]; p=0·0030) than Gram-negative organisms causing EOS. 43 (21·%) of 199 *K pneumoniae* isolates and 138 (86·3%) of 160 *A baumannii* isolates were resistant to carbapenems, with 98 (91·6%) of 107 *A baumannii* isolates causing inborn LOS being carbapenem resistant (appendix pp 10–11).

**Table 3:** Proportion of isolates causing neonatal bloodstream infection that are resistant to WHO-recommended antibiotics (n=809 isolates with antimicrobial or antifungal susceptibility results)

	All∗ isolates with resistance	EOS bloodstream infection	Late-onset bloodstream infection	Inborn LOS	Readmitted LOS
**Gram-negative organisms**					
Ampicillin and gentamicin	306/517 (59·2%)	54/136 (39·7%)	252/381 (66·1%)	224/325 (68·9%)	28/56 (50·0%)
Third-generation cephalosporins (ceftazidime)	325/491 (66·2%)	59/122 (48·4%)	266/369 (72·1%)	236/315 (74·9%)	30/54 (55·6%)
Piperacillin–tazobactam and amikacin	151/478 (31·6%)	28/116 (24·1%)	123/362 (34·0%)	117/314 (37·3%)	6/48 (12·5%)
Piperacillin–tazobactam	238/458 (52·0%)	37/111 (33·3%)	201/347 (57·9%)	185/299 (61·9%)	16/48 (33·3%)
Amikacin	66/389 (17·0%)	12/100 (12·0%)	54/289 (18·7%)	48/241 (19·9%)	6/48 (12·5%)
Carbapenems	192/493 (38·9%)	37/123 (30·1%)	155/370 (41·9%)	145/317 (45·7%)	10/53 (18·9%)
Imipenem	175/462 (37·9%)	31/113 (27·4%)	144/349 (41·3%)	136/301 (45·2%)	8/48 (16·7%)
Meropenem	184/491 (37·5%)	37/123 (30·1%)	147/368 (39·9%)	137/315 (43·5%)	10/53 (18·9%)
**Gram-positive organisms**					
Ampicillin and gentamicin	94/261 (36·0%)	20/105 (19·0%)	74/156 (47·4%)	65/111 (58·6%)	9/45 (20·0%)
Gentamicin	103/203 (50·7%)	23/65 (35·4%)	80/138 (58·0%)	71/105 (67·6%)	9/33 (27·3%)
Ampicillin	141/257 (54·9%)	42/106 (39·6%)	99/151 (65·6%)	78/107 (72·9%)	21/44 (47·7%)
Cloxacillin	32/85 (37·6%)	7/27 (25·9%)	25/58 (43·1%)	22/37 (59·5%)	3/21 (14·3%)
Vancomycin	3/252 (1·2%)	1/104 (1·0%)	2/148 (1·4%)	2/104 (1·9%)	0/44 (0·0%)
Linezolid	1/220 (0·5%)	0/88 (0·0%)	1/132 (0·8%)	0/95 (0·0%)	1/37 (2·7%)
**Fungal pathogens**					
Fluconazole	10/24 (41·7%)	2/7 (28·6%)	8/17 (47·1%)	6/15 (40·0%)	2/2 (100%)
Voriconazole	2/31 (6·5%)	0/8 (0·0%)	2/23 (8·7%)	1/21 (4·8%)	1/2 (50%)
Caspofungin	0/26	0/8	0/18	0/16	0/2

Data are n/N (%). EOS=early-onset sepsis (0–2 days of life). Inborn LOS HAI=late-onset sepsis hospital-associated infection among neonates who have never left the hospital since birth. LOS=late-onset sepsis (3–27 days of life). Readmitted LOS=late-onset sepsis among neonates admitted from home.

∗ Of all isolates available for antimicrobial or antifungal susceptibility testing.

94 (36·0%) of 261 Gram-positive organisms were resistant to both ampicillin–penicillin and gentamicin. Gram-positive organisms causing inborn LOS were six times more likely to be resistant to ampicillin–penicillin plus gentamicin combination therapy (OR 6·0 [95% CI 3·2–11·1]; p<0·0001) than those causing EOS (table 3). 32 (37·6%) of 85 *S aureus* isolates were methicillin-resistant (MRSA) and those with inborn LOS were four times more likely to have an MRSA infection than neonates with EOS (OR 4·3 [95% CI 1·3–14·4]; p=0·021; appendix pp 10–11). Only 1 (0·9%) of 110 of *Enterococcus* isolates were vancomycin resistant.

The prevalence of multidrug-resistant bacterial pathogens was 65·9% (504 of 765 cases) overall: 74·5% (413 of 554 cases) among Gram-negative pathogens and 56·9% (120 of 211 cases) among Gram-positive pathogens (appendix pp 10–11). Pathogens causing inborn LOS were almost four times more likely to be multidrug resistant (OR 3·5 [95% CI 2·5–5·1]; p<0·0001) than those causing EOS. Multidrug resistance was most frequent among neonates with *E faecium* (67 [90·5%] of 74 cases) infection, followed by *A baumannii* (151 [87·8%] of 172 cases) and *K pneumoniae* (167 [80·3%] of 208 cases) infection.

Of the fungal pathogens with available breakpoints for determination of antifungal susceptibility, ten (41·7%) of 24 isolates were resistant to fluconazole and two (6·5%) of 31 to voriconazole; no echinocandin resistance was observed. Two (28·6%) of seven fungal isolates causing EOS were resistant to fluconazole, whereas eight (47·1%) of 17 fungal isolates causing LOS were resistant to fluconazole and two (8·7%) of 23 to voriconazole.

Of 792 bloodstream infection episodes with clinical and mortality data, all-cause mortality by day 27 was 25·5% (202 of 792 cases), with 131 (64·9%) of 202 deaths attributable to the bloodstream infection (ie, occurred within 3 days of the specimen collection). Among all those who died, median time from hospital admission to death was 7 days (IQR 4–14), whereas those who survived the bloodstream infection episode spent a median of 20 days (IQR 11–37) in hospital (*vs* those who died in hospital; p<0·0001; table 1). Deaths (all causes) were four times more likely to occur among neonates with a bloodstream infection with Gram-negative versus Gram-positive pathogens (165 [32·8%] of 503 Gram-negative bloodstream infections *vs* 30 [12·0%] of 249 Gram-positive bloodstream infections resulted in death; OR 3·56 [95% CI 2·33–5·45]; p<0·0001; table 4). Seven (17·5%) of 40 neonates with a fungal bloodstream infection died (*vs* Gram-positive bloodstream infection, OR 1·55 [95% CI 0·63–3·81]; p=0·34). Overall, 49 (20·4%) of 240 neonates with EOS died versus 153 (27·7%) of 552 neonates with LOS (OR 1·62 [95% CI 1·12–2·36]; p=0·011). Other variables significantly associated with all-cause mortality by day 27 in the univariable analysis are shown in table 4.

**Table 4:** Multivariable analysis of risk factors for death after an episode of neonatal bloodstream infection (N=792 with outcome)

	Total number	Alive	Died	Univariable analysis: OR (95% CI)	p value	Multivariable analysis: adjusted OR (95% CI)	p value
	792	590 (74·5%)	202 (25·5%)	..	..	..	..
Hospital type							
Provincial	418	332 (79·4%)	86 (20·6%)	ref	..	..	..
Regional or district	374	258 (69·0%)	116 (31·0%)	1·74 (1·26–2·40)	0·0008	..	..
Gestational age, weeks (676 with data)							
Preterm	472	326 (69·1%)	146 (30·9%)	4·93 (2·89–8·40)	<0·0001	5·00 (2·16–11·59)	0·0002
Term	204	187 (91·7%)	17 (8·3%)	ref	..	ref	..
Presentation (timing of sepsis onset)							
EOS	240	191 (79·6%)	49 (20·4%)	ref	..	ref	..
Inborn LOS	449	317 (70·6%)	132 (29·4%)	1·62 (1·12–2·36)	0·011	2·42 (1·11–5·29)	0·027
Readmitted LOS	103	82 (79·6%)	21 (20·4%)	1·00 (0·56–1·77)	0·99	0·91 (0·24–3·40)	0·88
Admission to NICU							
Yes	436	303 (69·5%)	133 (30·5%)	1·83 (1·31–2·55)	0·0004	3·26 (1·51–7·03)	0·0026
No	356	287 (80·6%)	69 (19·4%)	ref	..	ref	..
Median age on date of specimen collection, days	5 (1–10)	6 (1–11)	5 (3–8)	0·98 (0·96–1·01)	0·14	..	..
Median time from specimen collection to outcome, days	10 (3–23)	14 (7–29)	1 (0–4)	0·86 (0·84–0·89)	<0·0001	0·79 (0·74–0·84)	<0·0001
Day 1 antibiotic treatment							
Ampicillin and gentamicin	139	116 (83·5%)	25 (18·0%)	ref	..	..	..
Piperacillin–tazobactam and amikacin	86	65 (75·6%)	21 (24·4%)	1·63 (0·84–3·17)	0·15	..	..
Meropenem	175	118 (67·4%)	57 (32·6%)	2·44 (1·41–4·21)	0·0014	..	..
Third-generation cephalosporin	39	35 (89·7%)	4 (10·3%)	0·58 (0·19–1·78)	0·34	..	..
Other	85	73 (85·9%)	12 (14·1%)	0·83 (0·39–1·77)	0·63	..	..
Pathogen type							
Gram-positive	249	219 (88·0%)	30 (12·0%)	ref	..	ref	..
Gram-negative	503	338 (67·2%)	165 (32·8%)	3·56 (2·33–5·45)	<0·0001	3·70 (1·46–9·39)	0·0059
Fungal	40	33 (82·5%)	7 (17·5%)	1·55 (0·63–3·81)	0·34	1	..
Ampicillin–penicillin plus gentamicin antimicrobial resistance (646 with data)							
No	313	243 (77·6%)	70 (22·4%)	ref	..	..	..
Yes	333	218 (65·5%)	115 (34·5%)	1·83 (1·29–2·60)	0·0007	..	..
No	279	187 (67·0%)	92 (33·0%)	ref	..	..	..
Yes	134	72 (53·7%)	62 (46·3%)	1·75 (1·15–2·67)	0·0092	..	..
Carbapenem antimicrobial resistance (418 with data)							
No	261	189 (72·4%)	72 (27·6%)	ref	..	..	..
Yes	167	84 (50·3%)	83 (49·7%)	2·59 (1·73–3·90)	<0·0001		..
Multidrug resistant organism (591 with data)							
No	203	163 (80·3%)	40 (19·7%)	ref	..	..	..
Yes	388	253 (65·3%)	135 (34·8%)	2·17 (1·45–3·26)	0·0002	..	..
Pathogen antimicrobial susceptibility to day 1 antibiotic received (359 with data)							
Concordant	242	189 (78·1%)	53 (21·9%)	ref	..	ref	..
Discordant	117	77 (65·8%)	40 (34·2%)	1·85 (1·14–3·02)	0·013	0·86 (0·40–1·88)	0·71
Pathogen							
*Staphylococcus aureus*	71	62 (87·3%)	9 (12·7%)	ref	..	..	..
*Enterococcus faecium*	49	45 (91·8%)	4 (8·2%)	0·61 (0·18–2·11)	0·43	..	..
*Enterococcus faecalis*	34	29 (85·3%)	5 (14·7%)	1·19 (0·37–3·86)	0·78	..	..
*Streptococcus agalactiae*	52	42 (80·8%)	10 (19·2%)	1·64 (0·61–4·38)	0·32	..	..
*Klebsiella pneumoniae*	205	139 (67·8%)	66 (32·2%)	3·27 (1·53–6·98)	0·0022	..	..
*Acinetobacter baumannii*	151	88 (58·3%)	63 (41·7%)	4·93 (2·28–10·66)	<0·0001	..	..
*Enterobacter cloacae*	42	28 (66·7%)	14 (33·3%)	3·44 (1·33–8·90)	0·011	..	..
*Escherichia coli*	29	21 (72·4%)	8 (27·6%)	2·62 (0·90–7·68)	0·078	..	..
*Serratia marcescens*	24	20 (83·3%)	4 (16·7%)	1·38 (0·38–4·96)	0·62	..	..
*Pseudomonas aeruginosa*	6	3 (50·0%)	3 (50·0%)	6·89 (1·20–39·50)	0·030	..	..
Other organisms	129	113 (87·6%)	16 (12·4%)	..	..	..	..

Data are n, n (%), or median (IQR) unless otherwise specified. Observations with missing data were excluded from the final multivariable analysis. EOS=early-onset sepsis (0–2 days of life). Inborn LOS=late-onset sepsis among neonates who have never left the hospital since birth. LOS=late-onset sepsis (3–27 days of life). NICU=neonatal intensive care unit. OR=odds ratio. Readmitted LOS=late-onset sepsis among neonates admitted from home.

In multivariable analysis, the following factors were associated with increased all-cause mortality: having a Gram-negative bloodstream infection (*vs* Gram-positive, adjusted OR 3·70 [95% CI 1·46–9·39]; p=0·0059), developing inborn LOS (*vs* EOS, 2·42 [1·11–5·29]; p=0·027), being preterm (5·00 [2·16–11·59]; p=0·0002), and being admitted to a neonatal ICU (3·26 [1·51–7·03]; p=0·0026; table 4). Most of the neonates with bloodstream infection who died, died on the first day after the positive blood culture specimen was taken; however, for each day beyond the positive culture, neonates had a 21% increased odds of survival (adjusted OR 0·79 [95% CI 0·74–0·84]; p<0·0001).

## Discussion

This study on neonatal bloodstream infection in lower-tier hospitals showed a dominance of Gram-negative pathogens. Most bloodstream infections were late-onset and occurred among preterm neonates or those with a very low birthweight, underscoring their vulnerability. A quarter of the neonates with bloodstream infection died (compared with 4% of hospitalised neonates without bloodstream infection), with highest mortality among those with Gram-negative pathogens. Five bacteria (*K pneumoniae, A baumannii, E faecium, S aureus*, and *S agalactiae*) accounted for two-thirds of infections, with the first three organisms showing 80% or higher multidrug resistance.

Blood culture positivity (6·8%) and pathogen yield (2·7%) were relatively low compared with other studies from southern Africa, which reported 12–22% culture positivity and 4–10% pathogen yield.^21^, ^22^, ^23^ These studies were conducted at large academic hospitals with more experienced staff.^7^^,^^22^, ^23^, ^24^ Difficulties in venepuncture and obtaining sufficient blood volume are notable in preterm and very low birthweight neonates.^25^ Contamination rates were consistent with other South African studies, mainly involving single coagulase-negative *Staphylococcus* spp of undetermined pathogenicity.^22^^,^^23^

In our study, 69% of neonates with bloodstream infection were preterm and of very low birthweight, often requiring long hospital stays. Bloodstream infection onset typically occurred on day 6 of life. These factors might explain the high proportion of LOS compared with EOS and the dominance of multidrug-resistant Gram-negative pathogens. Other studies from low-income and middle-income countries report similar rates of EOS and LOS; however, in many of these settings, births only occur in health-care facilities if complications are expected.^7^^,^^23^^,^^26^^,^^27^

The pathogen distribution of EOS was more varied than LOS with *K pneumoniae, S agalactiae, A baumannii*, and *S aureus* as the top four pathogens. *S agalactiae* was the most common EOS pathogen after *K pneumoniae*, but not as dominant as in Soweto, South Africa.^23^ EOS pathogens were less resistant than inborn LOS pathogens, although 40% of Gram-negative isolates were resistant to WHO first-line combination therapy and 30% to carbapenems. High multidrug-resistant *K pneumoniae* carriage in the birth canal or rapid colonisation of the neonate within hours of birth from the hospital environment might explain its presence in EOS.^28^^,^^29^ *S agalactiae* isolates were β-lactam susceptible and a fifth of *S aureus* isolates were methicillin resistant. Although WHO first-line therapy might be effective for Gram-positive EOS unless *Staphylococcus* infection is suspected, Gram-negative infections require a broader spectrum empirical regimen.

Our reported rate of inborn LOS was similar to a Cape Town central hospital study, with most infections caused by *K pneumoniae* and *A baumannii,* the latter less well described as a cause of LOS.^22^^,^^24^ Among all Gram-negative pathogens causing inborn LOS, more than two-thirds were resistant to WHO first-line therapy, 75% to third-generation cephalosporins, and 46% to carbapenems, with 92% carbapenem resistance demonstrated by *A baumannii* isolates, consistent with previous data.^7^^,^^22^^,^^30^^,^^31^ This finding underscores the need for enhanced infection prevention and control and antimicrobial stewardship.^22^^,^^31^^,^^32^

The lowest bloodstream infection rate was among neonates with community-acquired LOS. *E coli* was more likely to be cultured from these neonates. *K pneumoniae*, *S aureus*, and *S agalactiae* were also common pathogens, consistent with the ANISA study findings in south Asia.^6^

Although fungal pathogens represented only 5% of bloodstream infection episodes, many were multidrug-resistant isolates, including *C parapsilosis* and *C auris*. Large candidaemia outbreaks were previously reported in South Africa’s Gauteng province.^33^ The emergence of *C auris* in lower-tier units is very concerning.^34^ Triazole resistance among almost half of LOS *Candida* isolates limits empirical treatment to polyenes or echinocandins, which might not be available in lower-tier hospitals.^35^

Studies from low-income countries report increasing antimicrobial resistance among neonates with bloodstream infection.^24^^,^^36^ Our study supports this finding, with nearly two-thirds of bloodstream infection-causing organisms being multidrug resistant, including all presentations of bloodstream infection. *K pneumoniae, A baumannii*, and *E faecium*, which showed 80% or higher multidrug resistance, were among the top bacterial causes. Although only one LOS episode involved vancomycin-resistant *Enterococcus*, MRSA prevalence was 34%, in line with the declining trend in South African studies.^4^^,^^37^

The high mortality associated with neonatal bloodstream infection at lower-tier hospitals is notable, with one-quarter of neonates dying, accounting for over 20% of all deaths among hospitalised neonates. Many were preterm babies who acquired infections with multidrug-resistant pathogens by day 5 or day 6 of hospital admission. The combination of preterm birth, neonatal ICU admission, and Gram-negative bloodstream infection predicts a fatal outcome within the first week of life. Other studies have showed similar poor outcomes at central hospitals where babies might be sicker or require more care; however, infection prevention and control is suboptimal at all hospital levels.^5^^,^^8^^,^^27^

Our study’s setting in lower-tier hospitals in an upper-middle-income African country is unique. We collected clinical and laboratory data and confirmed pathogen identification and antimicrobial susceptibility. The results were similar to a national study.^4^ However, our study is not without limitations. Our laboratory-confirmed bloodstream infection definition probably underestimated the true incidence of invasive neonatal infections as many culture-negative bloodstream infection cases or those without blood cultures were excluded. A South African neonatal minimally invasive autopsy study demonstrated this selection bias, showing that almost 60% of all neonatal deaths had infection as an underlying or immediate cause of death.^38^ Another limitation is that our observational study used clinical data from medical records, therefore missing data occurred in multiple variables, including some used to control for confounding. These missing data were a combination of missing at random and missing completely at random; therefore, the observations were removed from the final multivariable analyses. Although we do not believe that this reduced the representativeness of the overall study, these missing data could have resulted in a reduction in statistical power of associations, or a loss of value from other data contributed by these observations.

Continued surveillance of neonatal bloodstream infection in lower-tier neonatal units, particularly with regard to low-birthweight and preterm infants with Gram-negative infections, is crucial as small vulnerable newborns (including preterm and small for gestational age babies) account for more than half of neonatal deaths.^39^ The high incidences of antimicrobial resistance highlight the need for infection control and antibiotic stewardship in neonatal units, with regular review of empirical antibiotic recommendations using local data. Unfortunately, most bloodstream infection-related deaths occur within 1 day of blood culture. Preventive measures, including clinician awareness of circulating pathogens, cleaning and sterilising of fomites, enforcement of handwashing, and adequate bed spacing, are essential to reduce infection spread in neonatal units.

## Data sharing

Should researchers request access to this data for further study or require additional information, they should communicate with the corresponding author. De-identified data will be made available on request once a protocol has been received and a data-access agreement has been signed between the National Institute for Communicable Diseases and the requestor.

## Declaration of interests

SM, CC, and AvG received grant funds from Sanofi Pasteur unrelated to this work. AvG has received grant funds from Center for Disease Control (Atlanta, USA) unrelated to this work. AvG is the chairperson of the National Advisory Group for Immunisation in South Africa. AD is supported by a National Institutes of Health Emerging Global Leader Award (NIH K43 TW010682) and is a member of the GARDP scientific advisory committee. NPG was partly supported by the US National Institutes of Health (1R01AI118511–01A1), UK National Institute for Health Care Research (Award ID: NIHR134342), and UK Medical Research Council (MR/V005731/10). NPG is the co-chair of the National Neonatal Sepsis Task Force. SW and CC have received funds from the Bill & Melinda Gates Foundation and the Center for Disease Control (Atlanta, USA) unrelated to this work. CC has also received grants from the Wellcome Trust and South African Medical Research Council unrelated to this work. All other authors declare no competing interests.
