# Supplementary material for: Pathogen aetiology and risk factors for death among neonates with bloodstream infections at lower-tier South African hospitals: a cross-sectional study
Source: Lancet Microbe. 2025 May;6(5):None. doi: 10.1016/j.lanmic.2024.100989 (PMC12062197; doi:10.1016/j.lanmic.2024.100989)
Supplement: Supplementary appendix [file mmc1.pdf]

THE LANCET
Microbe

Supplementary appendix

This appendix formed part of the original submission and has been peer reviewed.
We post it as supplied by the authors.

Supplement to: Meiring S, Quan V, Mashau R, et al. Pathogen aetiology and risk factors for death among neonates with bloodstream infections at lower-tier South African hospitals: a cross-sectional study. *Lancet Microbe* 2025. https://doi.org/10.1016/j.lanmic.2024.100989

Supplementary appendix

Title

Pathogen aetiology and risk factors for death among neonates with bloodstream infections at lower-tier South African hospitals: a cross-sectional study

Authors

Susan Meiring[1]; Vanessa Quan[1]; Rudzani Mashau[1]; Olga Perovic[1,2]; Rindidzani Magobo[1]; Marshagne Smith[1]; Ruth Mpembe[1], Anne von Gottberg[1,2,3]; Linda de Gouveia[1]; Sibongile Walaza[1]; Professor Cheryl Cohen[1]; Constance Kapongo[4]; Cheryl Mackay[5]; Mphekwa Thomas Mailula[6]; Omphile Mekgoe[7]; Lerato Motjale[8]; Rose Phayane[9]; Professor Angela Dramowski[10]; Professor Nelesh P. Govender[1,2,3,11] on behalf of Baby GERMS-SA

*A list of all other Baby GERMS-SA members can be found at the end of the manuscript

Affiliations

1. National Institute for Communicable Diseases, a Division of the National Health Laboratory Service, Johannesburg, South Africa
2. Faculty of Health Sciences, University of Witwatersrand, Johannesburg, South Africa
3. Faculty of Health Sciences, University of Cape Town, South Africa
4. Department of Paediatrics and Child Health, Queen Nandi Regional Hospital, Ngwelazane, South Africa
5. Department of Paediatrics and Child Health, Dora Nginza Hospital, Nelson Mandela Bay, South Africa
6. Department of Paediatrics and Child Health, Mankweng Regional Hospital, Mankweng, South Africa
7. Department of Paediatrics and Child Health, Klerksdorp Regional Hospital, Klerksdorp, South Africa
8. Department of Paediatrics and Child Health, Rob Ferreira Regional Hospital, Nelspruit, South Africa
9. Department of Paediatrics and Child Health, Tembisa Provincial Hospital, Johannesburg, South Africa
10. Department of Paediatrics and Child Health, Faculty of Medicine and Health Sciences, Stellenbosch University, Cape Town, South Africa
11. MRC Centre for Medical Mycology, University of Exeter, Exeter, United Kingdom

Table of Contents

Supplementary appendix 1

Title 1

Authors 1

Affiliations 1

Introduction 3

Supplementary methods 3

Isolate culture and identification 3

Statistical analysis 3

Ethics statement 4

Supplementary tables and figures: 4

Table S1: Characteristics of 6 lower-tier sentinel hospitals included as surveillance sites for culture-confirmed neonatal bloodstream-infections in South Africa 4

Table S2: Details of combination of organisms isolated in polymicrobial episodes of neonatal bloodstream infection (N=106) 6

Table S3a: List of discrepancies in identification of isolates identified from neonatal blood cultures 8

Table S3b: Comparison of antimicrobial susceptibility results generated by clinical diagnostic laboratories and the national reference laboratory 8

Table S4: Aetiology of bloodstream infection episodes by pathogen amongst HIV-exposed and -unexposed neonates (N=708) 9

Table S5: Phenotypic mechanisms of resistance for specific organisms causing neonatal blood stream infections (N=337 Gram-positive and N=628 Gram-negative organisms) 10

Table S6a: Multivariable analysis of risk factors for death following an episode of neonatal bloodstream infection caused by a Gram-negative bacterial organism (N=547 with outcome) 12

Table S6b: Multivariable analysis of risk factors for death following an episode of neonatal bloodstream infection caused by a Gram-positive bacterial organism (N=295 with outcome) 14

Table S7: Distribution of pathogens cultured from the cerebrospinal fluid of neonates admitted to 6 lower-tier hospitals, South Africa, 1 October 2019 through 30 September 2020 15

Figure S1a: Distribution of *Klebsiella pneumoniae* bloodstream infections occurring amongst neonates admitted at six lower-tier hospitals in South Africa by day of life, October 2019 through September 2020, N=233 16

Figure S1b: Distribution of *Acinetobacter baumannii* bloodstream infections occurring amongst neonates admitted at six lower-tier hospitals in South Africa by day of life, October 2019 through September 2020, N=174 16

Figure S1c: Distribution of *Staphylococcus aureus* bloodstream infections occurring amongst neonates admitted at six lower-tier hospitals in South Africa by day of life, October 2019 through September 2020, N=82 17

Figure S1d: Distribution of *Streptococcus agalactiae* bloodstream infections occurring amongst neonates admitted at six lower-tier hospitals in South Africa by day of life, October 2019 through September 2020, N=56 17

References: 17

Introduction

From 1 October 2019 through 30 September 2020, through the Baby GERMS-SA surveillance programme, we conducted a cross-sectional study among neonates with culture-confirmed sepsis admitted to 6 lower-tier sentinel hospitals. In addition to the 907 episodes of blood-stream infection, 26 episodes of culture-positive meningitis occurred amongst these neonates (Supplementary table 7, page 15). Some additional methods, analyses and results to supplement the main article are presented below.

Supplementary methods

Isolate culture and identification

Diagnostic microbiology laboratories all used automated blood culture systems. Bacterial and fungal pathogens were identified using Vitek-2 (bioMerieux, Marcy-l`Etoile, France), Microscan Walkaway (Beckman Coulter, Brea, CA, USA) or mass spectrometry instruments such as Vitek MS (bioMerieux, Marcy-l`Etoile, France). Antimicrobial susceptibility testing was performed using Vitek-2 or Microscan and interpreted according to the Clinical and Laboratory Standards Institute (CLSI) recommendations.[1] Five of the six diagnostic laboratories were accredited to ISO 15189:2012 by the South African National Accreditation System (SANAS), an independent body, and participated in a proficiency testing scheme.

At the NICD, identification of bacterial and fungal isolates was confirmed using phenotypic tests and/or a matrix-assisted laser desorption ionization of time of flight mass spectrometry (MALDI TOF-MS) Biotyper system (Bruker Daltonics GmbH, Bremen, Germany). For pathogenic organisms, minimum inhibitory concentrations (MICs) were generated on a MicroScan instrument (Beckman Coulter, Inc., West Sacramento, CA, USA) using Microscan GNX2F, Neg MIC Type 44 or Positive MIC Type 33 panels (Thermo Fisher Scientific), or manually using Sensititre STP6F (*Streptococcus* species) or YeastOne Y010 (*Candida* species) panels (Thermo Fisher Scientific). The following agents were tested: ampicillin, ampicillin/sulbactam, amoxicillin/clavulanic acid, amikacin, aztreonam, cefepime, cefotaxime, cefotaxime/K clavulanate, cefoxitin, ceftazidime, ceftazidime/K clavulanate, cefuroxime, cephalothin, chloramphenicol, ciprofloxacin, colistin, doripenem, ertapenem, imipenem, meropenem, fosfomycin, gentamicin, levofloxacin, minocycline, nitrofurantoin, norfloxacin, piperacillin/tazobactam, piperacillin, tetracycline, tobramycin, trimethoprim/sulphonamide, fluconazole, miconazole, voriconazole and caspofungin. MICs were interpreted according to the CLSI M100/ M27M44S (CLSI, 2021).[1] ATCC strains were included in quality control (QC) runs on all days of testing and MICs were consistently found to be within acceptable QC ranges. Isolates were classified as multidrug-resistant (MDR) if they were non-susceptible to ≥1 agent in ≥2 antimicrobial classes as defined by an international expert committee.[2]

In addition, culture-confirmed pathogens from cerebrospinal fluid specimens were reported and isolates were submitted to the NICD. These are only reported in the supplementary material (Supplementary Table 7, page 15).

Statistical analysis

All statistical analyses were performed using STATA statistical software version 17 (StataCorp Inc., College Station, TX, USA). Descriptive statistics were reported as frequencies for categorical variables and medians with interquartile ranges for continuous variables. The rate of blood culture specimen collection was calculated using number of blood cultures taken during the study period divided by the total number of neonates admitted

to the six hospitals during the same period. The incidence rate of BSI was expressed as cases per 1000 patient-days (calculated by dividing the number of episodes by the total number of patient days (i.e. sum of the neonates admitted each day in each hospital)), as well as cases per 1000 live births (using a denominator of live births registered in the 6 districts served by each hospital).The in-hospital mortality risk was calculated as the number of neonatal deaths occurring in hospital divided by the total number of neonates admitted during the study period. The crude case-fatality ratio amongst neonates with a BSI was calculated as a percentage of deaths during the neonatal period amongst those with BSI. Attributable mortality was calculated as the number of deaths within 3 days of the sepsis episode divided by the total number of neonates with BSI. Univariate analysis was performed using Fisher's exact/ chi-square test for each categorical variable and in-hospital outcome (alive or dead by discharge or day 27 of life (if still admitted)). Three variables were included *a priori* in the models, these included gestational age category, admission to an ICU and empiric antibiotic therapy concordant with organism isolated. In addition, variables with a p-value of <0·2 on univariate analysis were included in all multivariable logistic regression models, and those with a p-value of >0·05 were then dropped using step-wise manual backward elimination. Separate multivariable logistic regression models for risk factors associated with death are reported by Gram-negative and Gram-positive pathogens (Supplementary tables 6a and 6b, pages 12-14).

Ethics statement

The study was approved by the Human Research Ethics Committee of the University of the Witwatersrand (M190320). Approvals for this surveillance study were received from each provincial research committee through registration on the National Health Research Database.

Supplementary tables and figures:

Table S1: Characteristics of 6 lower-tier sentinel hospitals included as surveillance sites for culture-confirmed neonatal bloodstream-infections in South Africa

	Hospital Name					
	**Rob Ferreira**	**Mankweng**	**Klerksdorp**	**Dora Nginza**	**Queen Nandi**	**Tembisa**
**Province**	Mpumalanga	Limpopo	North West	Eastern Cape	KwaZulu-Natal	Gauteng
**District**	Ehlanzeni	Capricorn	Dr Kenneth Kaunda	Nelson Mandela Metropolitan	Uthungulu	Ekurhuleni Metropolitan
**Level**	Provincial	Provincial	Provincial	Regional	Regional	Regional
**Live births registered within the district in 2019***	41067	30401	13206	17481	18838	62421
**Obstetric care**						
Maternity Intensive Care Unit	YES	YES	YES	NO	NO	NO
High and low risk pregnancies	YES	YES	YES	YES	YES	YES
**Neonatal services**						
Intensive Care Beds	YES	YES	YES	YES	YES	YES
High Care Beds	YES	YES	YES	YES	YES	YES
Kangaroo Care Beds	YES	YES	YES	YES	YES	YES

**Microbiology services**						
Microbiology laboratory onsite	YES	YES	YES	NO	NO	NO
Pathologist onsite	YES	NO	NO	NO	NO	NO
**Infection Prevention and Control services**						
IPC nurse in hospital	YES	YES	YES	YES	YES	YES
IPC nurse assigned to neonatal unit	NO	NO	NO	NO	YES	YES
**Pharmacy services**						
Pharmacist in the hospital	YES	YES	YES	YES	YES	YES
Pharmacist assigned to neonatal unit	YES	NO	NO	YES	YES	YES

*Registered live births available from Stats SA, Recorded Live Births P0305, page 41,42 (https://www.statssa.gov.za/publications/P0305/P03052019.pdf)

Table S2: Details of combination of organisms isolated in polymicrobial episodes of neonatal bloodstream infection (N=106)

Number of Episodes Reported	Organism 1	Organism 2	Organism 3	Organism 4	Organism 5
1	*Enterococcus faecalis*	Coagulase negative *Staphylococcus*	*Serratia marcescens*	*Candida* species	*Stenotrophomonas maltophilia*
1	*Acinetobacter baumannii*	*Klebsiella pneumoniae*	*Enterococcus faecium*	*Candida* species	
1	*Acinetobacter baumannii*	*Klebsiella pneumoniae*	*Candida* species		
1	*Staphylococcus aureus*	*Serratia marcescens*	*Candida* species		
1	*Acinetobacter baumannii*	*Staphylococcus aureus*	Coagulase negative *Staphylococcus*		
1	*Acinetobacter baumannii*	*Enterobacter cloacae* complex	*Enterococcus faecalis*		
1	*Acinetobacter baumannii*	*Klebsiella pneumoniae*	*Enterococcus faecalis*		
1	*Acinetobacter baumannii*	*Enterobacter cloacae* complex	*Enterococcus faecium*		
1	*Acinetobacter baumannii*	*Klebsiella pneumoniae*	*Enterococcus faecium*		
1	*Acinetobacter baumannii*	*Staphylococcus aureus*	*Enterococcus faecium*		
1	*Klebsiella pneumoniae*	*Enterococcus faecium*	*Escherichia coli*		
9	*Acinetobacter baumannii*	*Enterococcus faecium*			
3	*Acinetobacter baumannii*	*Candida* species			
3	*Acinetobacter baumannii*	*Enterobacter cloacae* complex			
3	*Acinetobacter baumannii*	*Enterococcus faecalis*			
2	*Acinetobacter baumannii*	*Staphylococcus aureus*			
1	*Acinetobacter baumannii*	*Escherichia coli*			
1	*Acinetobacter baumannii*	*Serratia marcescens*			
2	*Candida species*	Coagulase negative *Staphylococcus*			
2	*Enterobacter cloacae* complex	*Staphylococcus aureus*			
1	*Enterobacter cloacae* complex	*Lactococcus lactis*			
1	*Enterococcus faecalis*	*Enterococcus faecium*			
1	*Enterococcus faecalis*	*Serratia marcescens*			
1	*Enterococcus faecalis*	*Staphylococcus aureus*			

2	*Enterococcus faecium*	*Enterobacter cloacae* complex
1	*Enterococcus faecium*	*Candida* species
1	*Enterococcus faecium*	*Enterobacter aerogenes*
1	*Enterococcus faecium*	*Morganella morganii*
1	*Enterococcus faecium*	*Proteus mirabilis*
1	*Enterococcus faecium*	*Serratia marcescens*
1	*Klebsiella oxytoca*	*Enterobacter cloacae* complex
12	*Klebsiella pneumoniae*	*Acinetobacter baumannii*
10	*Klebsiella pneumoniae*	*Enterobacter cloacae* complex
10	*Klebsiella pneumoniae*	*Enterococcus faecium*
8	*Klebsiella pneumoniae*	*Enterococcus faecalis*
5	*Klebsiella pneumoniae*	*Candida* species
3	*Klebsiella pneumoniae*	*Serratia marcescens*
2	*Klebsiella pneumoniae*	*Escherichia coli*
2	*Klebsiella pneumoniae*	*Staphylococcus aureus*
1	*Klebsiella pneumoniae*	*Proteus mirabilis*
1	*Klebsiella pneumoniae*	*Pseudomonas aeruginosa*
2	*Streptococcus agalactiae*	*Staphylococcus aureus*
1	*Streptococcus pneumoniae*	*Escherichia coli*

Table S3a: List of discrepancies in identification of isolates identified from neonatal blood cultures

NICD identification	Clinical lab identification
Enterococcus faecalis	*Acinetobacter baumannii*
Enterococcus faecalis	*Streptococcus* species
Staphylococcus aureus	*Enterococcus* species
Staphylococcus aureus	*Morganella morganii*
Staphylococcus epidermidis	*Acinetobacter baumannii*
Pseudomonas species	*Acinetobacter baumannii*
Lodderomyces elongisporus	*Candida* species
Wickerhamomyces anomalus	*Candida* species

Table S3b: Comparison of antimicrobial susceptibility results generated by clinical diagnostic laboratories and the national reference laboratory

		Clinical laboratory (only those sent to reference laboratory)			Reference laboratory						
	N	% Resistant	95% Confidence Interval	Standard error	% Resistant	95% Confidence Interval	Standard error	Standard error for difference	Difference in proportions	95% Confidence interval for difference	
**Methicillin resistant *Staphylococcus aureus***										(Lower limit)	(Upper limit)
Staphylococcus aureus	36	0·2778	0.1530-0.4501	0·0747	0·3261	0·2043-0·4769	0·0691	0·1017	0·0483	-0·0534	0·1500
**ESBL producing Enterobacterales (cefotaxime R)**											
All Enterobacterales	194	0·7487	0.6819-0.8054	0·0314	0·7655	0·7055-0·8164	0·0282	0·0422	0·0168	-0·0254	0·0590
**Vancomycin resistant Enterococcus**											
All enterococci	53	0·0182	0.0024-0.1229	0·0180	0·0448	0·0142-0·1323	0·0448	0·0483	0·0266	-0·0217	0·0749
**Carbapenem resistant Enterobacterales**											
All Enterobacterales	210	0·1981	0.1490-0.2583	0·0277	0·2531	0·2023-0·3115	0·0278	0·0392	0·055	0·0158	0·0942
Klebsiella pneumoniae	140	0·2721	0.2034-0.3536	0·0382	0·3494	0·2803-0·4255	0·037	0·0532	0·0773	0·0241	0·1305
**Carbapenem resistant *Acinetobacter***											
Acinetobacter baumannii	84	0·8966	0.8115-0.9458	0·0327	0·949	0·8821-0·9788	0·0222	0·0395	0·0524	0·0129	0·0919

Table S4: Aetiology of bloodstream infection episodes by pathogen amongst HIV-exposed and -unexposed neonates (N=708)

	All		EOS		LOS inborn		LOS readmitted	
**Top five Gram-negative pathogens**	**HIV Exposed**	**HIV unexposed**	**HIV Exposed**	**HIV unexposed**	**HIV Exposed**	**HIV unexposed**	**HIV Exposed**	**HIV unexposed**
	**n/N (%)**	**n/N (%)**	**n/N (%)**	**n/N (%)**	**n/N (%)**	**n/N (%)**	**n/N (%)**	**n/N (%)**
Klebsiella pneumoniae	67/162 (41)	114/285 (40)	13/35 (37)	19/81 (23)	48/113 (42)	87/176 (49)	6/14 (43)	8/28 (29)
Acinetobacter baumannii	53/162 (33)	76/285 (27)	7/35 (20)	20/81 (25)	44/113 (39)	49/176 (28)	2/14 (14)	7/28 (25)
Enterobacter cloacae	12/162 (7)	28/285 (10)	2/35 (6)	11/81 (14)	10/113 (9)	14/176 (8)	0/14 (0)	3/28 (11)
Escherichia coli	7/162 (4)	18/285 (6)	4/35 (11)	7/81 (9)	2/113 (2)	5/176 (3)	1/14 (7)	6/28 (21)
Serratia marcescens	8/162 (5)	16/285 (6)	2/35 (6)	4/81 (5)	5/113 (4)	11/176 (6)	1/14 (7)	1/28 (4)
**Other Gram-negative organisms**	15/162 (9)	33/285 (12)	7/35 (20)	20/81 (25)	4/113 (4)	10/176 (6)	4/14 (29)	3/28 (11)

	All		EOS		LOS inborn		LOS readmitted	
**Top five Gram-positive pathogens**	**HIV Exposed**	**HIV unexposed**	**HIV Exposed**	**HIV unexposed**	**HIV Exposed**	**HIV unexposed**	**HIV Exposed**	**HIV unexposed**
	**n/N (%)**	**n/N (%)**	**n/N (%)**	**n/N (%)**	**n/N (%)**	**n/N (%)**	**n/N (%)**	**n/N (%)**
Staphylococcus aureus	21/67 (31)	45/161 (28)	6/28 (21)	16/67 (24)	12/26 (46)	17/64 (27)	3/13 (23)	12/30 (40)
Streptococcus agalactiae	15/67 (22)	39/161 (24)	9/28 (32)	23/67 (34)	1/26 (4)	6/64 (9)	5/13 (38)	10/30 (33)
Enterococcus faecium	14/67 (21)	32/161 (20)	7/28 (25)	8/67 (12)	4/26 (15)	23/64 (36)	3/13 (23)	1/30 (3)
Enterococcus faecalis	9/67 (13)	21/161 (13)	3/28 (11)	11/67 (16)	5/26 (19)	6/64 (9)	1/13 (8)	4/30 (13)
Coagulase negative *Staphylococcus*	4/67 (6)	11/161 (7)	1/28 (4)	2/67 (3)	3/26 (12)	7/64 (11)	0/13 (0)	2/30 (7)
**Other Gram-positive organisms**	4/67 (6)	13/161 (8)	2/28 (7)	7/67 (10)	1/26 (4)	5/64 (8)	1/13 (8)	1/30 (3)

	All		EOS		LOS inborn		LOS readmitted	
**Top five fungal pathogens**	**HIV Exposed**	**HIV unexposed**	**HIV Exposed**	**HIV unexposed**	**HIV Exposed**	**HIV unexposed**	**HIV Exposed**	**HIV unexposed**
	**n/N (%)**	**n/N (%)**	**n/N (%)**	**n/N (%)**	**n/N (%)**	**n/N (%)**	**n/N (%)**	**n/N (%)**
Candida parapsilosis	3/11 (27)	11/22 (50)	1/2 (50)	7/9 (78)	2/9 (22)	4/13 (31)	0/0 (0)	0/0 (0)
Candida auris (C. haemulonii)	2/11 (18)	4/22 (18)	0/2 (0)	0/9 (0)	2/9 (22)	4/13 (31)	0/0 (0)	0/0 (0)
Candida albicans	3/11 (27)	2/22 (9)	1/2 (50)	1/9 (11)	2/9 (22)	1/13 (8)	0/0 (0)	0/0 (0)
Candida famata	1/11 (9)	2/22 (9)	0/2 (0)	0/9 (0)	1/9 (11)	2/13 (15)	0/0 (0)	0/0 (0)
Candida tropicalis	1/11 (9)	1/22 (5)	0/2 (0)	0/9 (0)	1/9 (11)	1/13 (8)	0/0 (0)	0/0 (0)
**Other fungal infections**	1/11 (9)	2/22 (9)	0/2 (0)	1/9 (11)	1/9 (11)	1/13 (8)	0/0 (0)	0/0 (0)

Footnote: EOS: Early-onset sepsis (0-2 days); LOS: Late-onset sepsis (3-27 days); LOS inborn: LOS in neonate admitted since birth; LOS Readmitted: LOS in neonate admitted from the community

Table S5: Phenotypic mechanisms of resistance for specific organisms causing neonatal blood stream infections (N=337 Gram-positive and N=628 Gram-negative organisms)

	N	Susceptible n (%)	Intermediate n (%)	Resistant n (%)
**Methicillin resistant *Staphylococcus aureus***				
Staphylococcus aureus	74	49 (66)	0 (0)	25 (34)
EOS	24	19 (79)	0 (0)	5 (21)
LOS inborn	32	15 (47)	0 (0)	17 (53)
LOS readmitted	18	15 (83)	0 (0)	3 (17)
**ESBL producing Enterobacteriaceae (cefotaxime resistant)**				
All	334	125 (37)	1 (1)	208 (62)
EOS	78	45 (58)	0 (0)	33 (42)
LOS inborn	199	59 (30)	1 (0)	139 (70)
LOS readmitted	39	16 (41)	0 (0)	23 (59)
**Vancomycin resistant *Enterococcus***				
All enterococci	110	109 (99)	0 (0)	1 (1)
EOS	40	40 (100)	0 (0)	0 (0)
LOS inborn	59	58 (98)	0 (0)	1 (2)
LOS readmitted	11	11 (100)	0 (0)	0 (0)
Enterococcus faecalis	43	43 (100)	0 (0)	0 (0)
Enterococcus faecium	70	70 (100)	0 (0)	0 (0)
Enterococcus species	2	1 (50)	0 (0)	1 (50)
**Carbapenem resistant *Pseudomonas***				
Pseudomonas aeruginosa	7	6 (86)	0 (0)	1 (14)
*Pseudomonas*species	4	4 (100)	0 (0)	0 (0)
**Carbapenem resistant Enterobacter**				
All	334	277 (83)	8 (2)	49 (15)
Klebsiella pneumoniae	199	150 (75)	6 (3)	43 (22)
EOS	33	25 (76)	1 (3)	7 (21)
LOS inborn	134	96 (72)	5 (3)	33 (25)
LOS readmitted	19	16 (84)	0 (0)	3 (16)
Klebsiella species	4	4 (100)	0 (0)	0 (0)
Enterobacter species	59	55 (93)	1 (2)	3 (5)
Morganella morganii	2	2 (100)	0 (0)	0 (0)
Proteus species	3	3 (100)	0 (0)	0 (0)
Serratia marcescens	28	24 (86)	1 (3)	3 (11)
Escherichia coli	36	36 (100)	0 (0)	0 (0)
**Carbapenem resistant *Acinetobacter***				
Acinetobacter baumannii	160	22 (14)	0 (0)	138 (86)
EOS	28	5 (18)	0 (0)	23 (82)
LOS inborn	107	9 (8)	0 (0)	98 (92)
LOS readmitted	12	5 (42)	0 (0)	7 (58)
Acinetobacter species	8	6 (75)	0 (0)	2 (25)
**Multidrug resistant pathogens**				
All	765			504 (66)

By presentation		
EOS	200	97 (49)
LOS inborn	429	329 (77)
LOS readmitted	88	42 (48)
All Gram-negative	554	413 (75)
Klebsiella pneumoniae	208	167 (80)
Acinetobacter baumannii	172	151 (88)
Enterobacter cloacae	58	37 (64)
All Gram-positive	211	120 (57)
Enterococcus faecium	74	67 (91)
Enterococcus faecalis	48	5 (10)
Staphylococcus aureus	75	25 (33)

Footnote: EOS: Early-onset sepsis (0-2 days); LOS: Late-onset sepsis (3-27 days)

Table S6a: Multivariable analysis of risk factors for death following an episode of neonatal bloodstream infection caused by a Gram-negative bacterial organism (N=547 with outcome)

	Total	Alive	Died	Univariable analysis		Multivariable analysis	
	N	n (%)	n (%)	Odds ratio (95% Confidence interval)	P-value	Odds ratio (95% Confidence interval)	P-value
	547	360 (66)	187 (34)				
**Ampicillin/penicillin plus gentamicin resistance**							
No	197	138 (70)	59 (30)	ref			
Yes	288	170 (59)	118 (41)	1·62 (1·10-2·39)	0·014		
**Piperacillin-tazobactam plus amikacin resistance**							
No	308	203 (66)	105 (34)	ref			
Yes	140	72 (51)	68 (49)	1·83 (1·21-2·74)	0·004		
**Carbapenem resistance**							
No	283	202 (71)	81 (29)	ref			
Yes	179	86 (48)	93 (52)	2·70 (1·83-3·98)	<0·001		
**Multidrug resistant organism**							
No	125	90 (72)	35 (28)	ref		ref	
Yes	360	217 (60)	143 (40)	1·69 (1·09-2·64)	0·02	0·57 (0·24-1·38)	0·215
**Presentation (timing of sepsis onset)**							
Early onset sepsis	134	100 (75)	34 (25)	ref		ref	
Inborn late onset sepsis	353	217 (61)	136 (39)	1·84 (1·18-2·87)	0·007	2·01 (0·68-6·00)	0·209
Readmitted late onset sepsis	60	43 (72)	17 (28)	1·16 (0·59-2·30)	0·665	1·80 (0·38-8·62)	0·459
**Median time in days from specimen collection to outcome**	11 (2-26)	19 (10-31)	1 (0-4)	0·85 (0·83-0·88)	<0·001	0·79 (0·74-0·84)	<0·001
**Median age in days on date of specimen collection**		6 (2-11)	5 (3-9)	0·99 (0·96-1·02)	0·413	1·00 (0·92-1·09)	0·983
**Hospital type**							
Regional/ District	267	191 (72)	76 (28)	ref			
Provincial	280	169 (60)	111 (40)	1·65 (1·15-2·36)	0·006		
**NICU admission**							
Yes	330	202 (61)	128 (39)	1·70 (1·17-2·46)	0·005	3·45 (1·56-7·63)	0·002
No	217	158 (73)	59 (27)	ref		ref	
**Day 1 antibiotic treatment**							
Ampicillin/penicillin plus gentamicin	86	69 (80)	17 (20)	ref		ref	
Piperacillin-tazobactam plus amikacin	62	41 (66)	21 (34)	2·08 (0·98-4·39)	0·055	2·27 (0·67-7·73)	0·188
Meropenem	149	90 (60)	59 (40)	2·66 (1·43-4·97)	0·002	2·90 (0·99-8·47)	0·052
Third generation cephalosporin	28	23 (82)	5 (18)	0·88 (0·29-2·66)	0·824	0·79 (0·14-4·36)	0·783
Other	49	38 (78)	11 (22)	1·17 (0·50-2·76)	0·712	0·81 (0·22-2·95)	0·75
**Gestational age**							
Preterm	354	220 (62)	134 (38)	3·25 (1·88-5·62)	<0·001	3·36 (1·46-7·71)	0·004
Term	114	96 (84)	18 (16)				
**Pathogen**	547	360 (66)	187 (34)				
Klebsiella pneumoniae	211	143 (68)	68 (32)	1·95 (0·95-4·0)	0·07		
Acinetobacter baumannii	160	91 (57)	69 (43)	3·10 (1·50-6·43)	0·002		

Enterobacter cloacae	57	32 (56)	25 (44)	3·20 (1·38-7·42)	0·007
Escherichia coli	34	24 (71)	10 (29)	1·70 (0·63-4·58)	0·291
Serratia marcescens	29	25 (86)	4 (14)	0·65 (0·19-2·27)	0·504
Other	56	45 (80)	11 (20)	ref	
**Pathogen/day 1 antibiotic received**	287	192 (67)	95 (33)		
Concordant	165	118 (72)	47 (28)		
Discordant	122	74 (61)	48 (39)	1·63 (0·99-2·67)	0·054

Table S6b: Multivariable analysis of risk factors for death following an episode of neonatal bloodstream infection caused by a Gram-positive bacterial organism (N=295 with outcome)

	Total	Alive	Died	Univariable analysis		Multivariable analysis	
	N	n (%)	n (%)	Odds ratio (95% Confidence interval)	P-value	Odds ratio (95% Confidence interval)	P-value
	295	252 (85)	43 (15)				
**Ampicillin/penicillin plus gentamicin resistance**	239	200 (84)	39 (16)				
No	150	128 (85)	22 (15)	ref			
Yes	89	72 (81)	17 (19)	1·37 (0·69-2·75)	0·371		
**Multidrug resistant organism**	183	155 (85)	28 (15)				
No	107	93 (87)	14 (13)	ref			
Yes	76	62 (82)	14 (18)	1·50 (0·67-3·36)	0·325		
**Presentation (timing of sepsis onset)**							
Early onset sepsis	114	94 (82)	20 (18)	1·25 (0·63-2·49)	0·517	3·41 (0·39-29·60)	0·267
Inborn late onset sepsis	131	112 (85)	19 (15)	ref		ref	
Readmitted late onset sepsis	50	46 (92)	4 (8)	0·51 (0·17-1·59)	0·247	1·	
**Admission to NICU**							
Yes	142	115 (81)	27 (19)	2·01 (1·03-3·91)	0·04	5·91 (1·30-26·84)	0·021
No	153	137 (90)	16 (10)	ref		ref	
**Median time in days from specimen collection to outcome (IQR)**	8 (3-19)	10 (5-21)	1 (1-4)	0·82 (0·76-0·89)	<0·001	0·81 (0·72-0·91)	<0·001
**Hospital type**							
District	180	157 (87)	23 (13)	ref			
Regional	115	95 (83)	20 (17)	1·44 (0·75-2·76)	0·275		
**Day 1 antibiotic treatment**	185	162 (88)	23 (12)				
Ampicillin/penicillin plus gentamicin	53	48 (91)	5 (9)	ref		ref	
Piperacillin-tazobactam plus amikacin	32	30 (94)	2 (6)	0·64 (0·12-3·51)	0·607	1·37 (0·10-19·61)	0·814
Carbapenem	48	35 (73)	13 (27)	3·57 (1·16-10·92)	0·026	7·39 (0·73-74·89)	0·09
Third generation cephalosporin	14	14 (100)	0 (0)	1·		1·	
Other	38	35 (92)	3 (8)	0·82 (0·18-3·67)	0·798	1·47 (0·27-8·16)	0·656
**Gestational age**	250	217 (87)	33 (13)				
Preterm	146	117 (80)	29 (20)	6·2 (2·11-18·23)	0·001	8·24 (1·73-39·12)	0·008
Term	104	100 (96)	4 (4)	ref		ref	
**Pathogen**							
Staphylococcus aureus	77	67 (87)	10 (13)	1·49 (0·30-7·38)	0·623		
Enterococcus faecium	74	61 (82)	13 (18)	2·13 (0·44-10·26)	0·345		
Streptococcus agalactiae	52	42 (81)	10 (19)	2·38 (0·48-11·90)	0·291		
Enterococcus faecalis	47	39 (83)	8 (17)	2·05 (0·40-10·58)	0·391		
Coagulase negative *Staphylococcus*	23	23 (100)	0 (0)	1·			
Other	22	20 (91)	2 (9)	ref			
**Pathogen/day 1 antibiotic received**	113						
Concordant	99	87 (88)	12 (12)	ref			
Discordant	14	12 (86)	2 (14)	1·21 (0·24-6·07)	0·818		

Table S7: Distribution of pathogens cultured from the cerebrospinal fluid of neonates admitted to 6 lower-tier hospitals, South Africa, 1 October 2019 through 30 September 2020

Pathogen	n/N (%)
**Gram-negative**	**15/26 (57·6)**
Enterobacter cloacae complex	5/26 (19·2)
Acinetobacter baumannii	3/26 (11·5)
Klebsiella pneumoniae	3/26 (11·5)
Serratia marcescens	3/26 (11·5)
Salmonella Group D	1/26 (3·9)
**Gram-positive**	**11/26 (42·4)**
Streptococcus agalactiae	8/26 (30·8)
Enterococcus faecium	2/26 (7·7)
Staphylococcus aureus	1/26 (3·9)

Footnote: 38 CSFs, 9 duplicate organisms, 5 polymicrobial episodes of which 2 are indicated in the table above (one *Klebsiella pneumoniae* and *Serratia marcescens on CSF*) (2 *Klebsiella* sp. on B/C and *A. baumannii* on CSF, one *Klebsiella* sp. on CSF and *Proteus* sp. on CSF, one *Serratia marcescens* on blood and CSF)

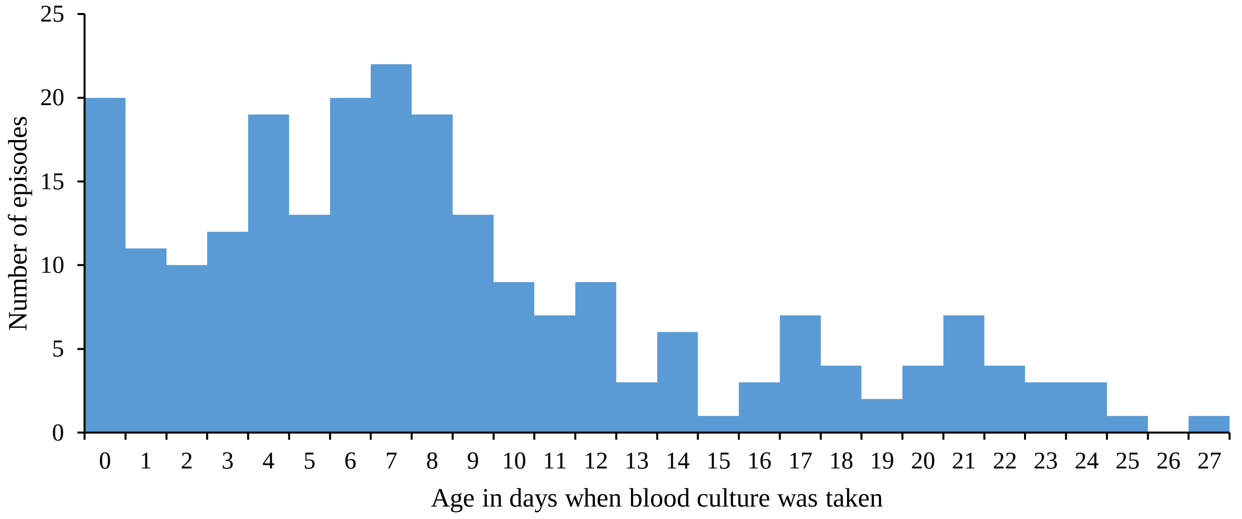

Figure S1a: Distribution of *Klebsiella pneumoniae* bloodstream infections occurring amongst neonates admitted at six lower-tier hospitals in South Africa by day of life, October 2019 through September 2020, N=233

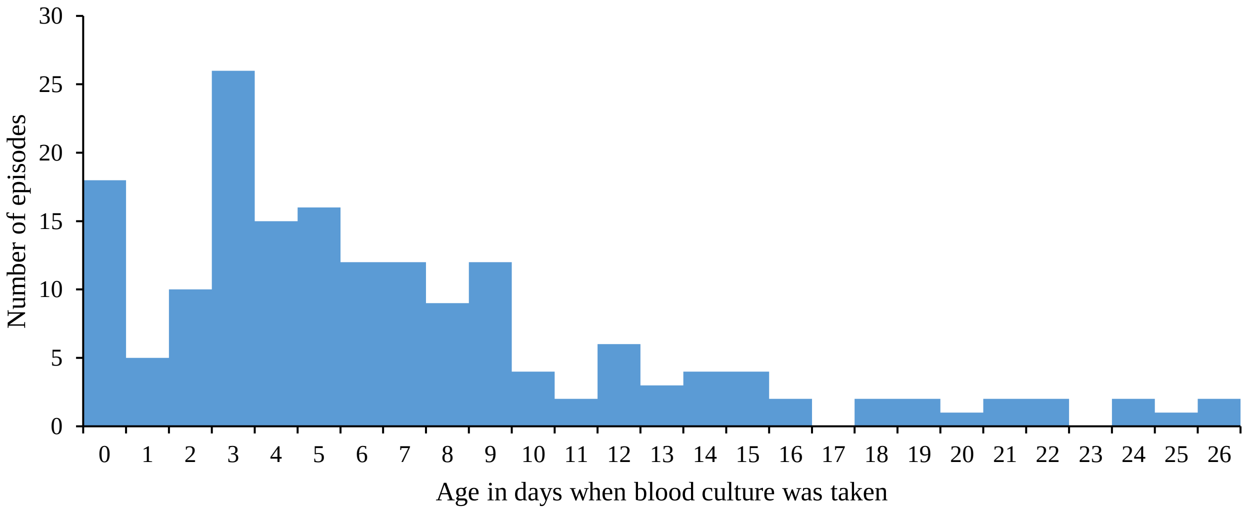

Figure S1b: Distribution of *Acinetobacter baumannii* bloodstream infections occurring amongst neonates admitted at six lower-tier hospitals in South Africa by day of life, October 2019 through September 2020, N=174

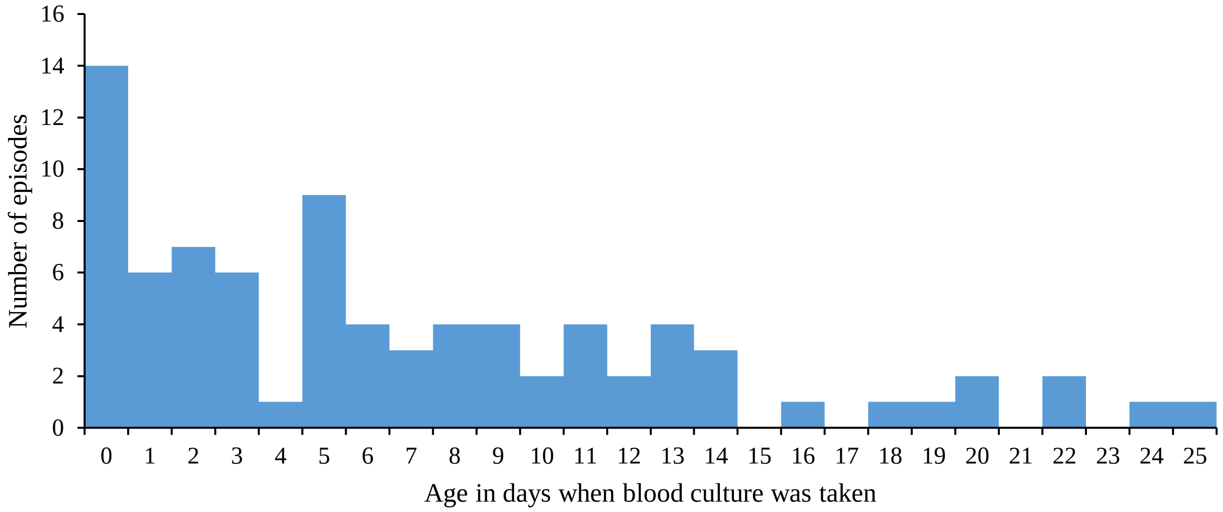

Figure S1c: Distribution of *Staphylococcus aureus* bloodstream infections occurring amongst neonates admitted at six lower-tier hospitals in South Africa by day of life, October 2019 through September 2020, N=82

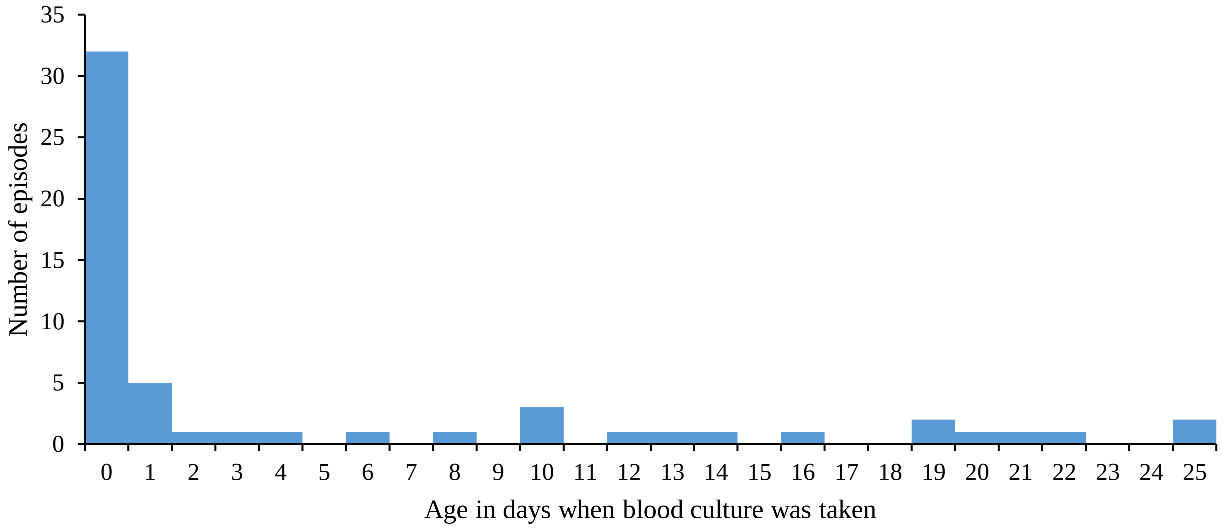

Figure S1d: Distribution of *Streptococcus agalactiae* bloodstream infections occurring amongst neonates admitted at six lower-tier hospitals in South Africa by day of life, October 2019 through September 2020, N=56

References:

1 Wayne P. CLSI. Performance Standards for Antimicrobial Susceptibility Testing. *CLSI Suppl M100* 2020; 30th Ed.

2 Magiorakos A, Srinivasan A, Carey RB, *et al.* Multidrug-resistant, extensively drug-resistant and pandrug-resistant bacteria : an international expert proposal for interim standard definitions for acquired resistance. *Clin Microbiol Infect* 2011; 18: 268–81.
